# Heterogeneity: The key to failure forecasting

**DOI:** 10.1038/srep13259

**Published:** 2015-08-26

**Authors:** Jérémie Vasseur, Fabian B. Wadsworth, Yan Lavallée, Andrew F. Bell, Ian G. Main, Donald B. Dingwell

**Affiliations:** 1 Earth and Environmental Sciences, Ludwig Maximilian University, Munich, Germany; 2 Earth, Ocean and Ecological Sciences, University of Liverpool, Liverpool, United Kingdom; 3 School of Geosciences, University of Edinburgh, Edinburgh, United Kingdom

## Abstract

Elastic waves are generated when brittle materials are subjected to increasing strain. Their number and energy increase non-linearly, ending in a system-sized catastrophic failure event. Accelerating rates of geophysical signals (*e.g.*, seismicity and deformation) preceding large-scale dynamic failure can serve as proxies for damage accumulation in the Failure Forecast Method (FFM). Here we test the hypothesis that the style and mechanisms of deformation, and the accuracy of the FFM, are both tightly controlled by the degree of microstructural heterogeneity of the material under stress. We generate a suite of synthetic samples with variable heterogeneity, controlled by the gas volume fraction. We experimentally demonstrate that the accuracy of failure prediction increases drastically with the degree of material heterogeneity. These results have significant implications in a broad range of material-based disciplines for which failure forecasting is of central importance. In particular, the FFM has been used with only variable success to forecast failure scenarios both in the field (volcanic eruptions and landslides) and in the laboratory (rock and magma failure). Our results show that this variability may be explained, and the reliability and accuracy of forecast quantified significantly improved, by accounting for material heterogeneity as a first-order control on forecasting power.

Most Earth materials exhibit significant structural heterogeneities. Common examples are local density fluctuations, pores, cracks and crystals^1^. The presence of these so-called Griffith flaws in materials provides sites of stress concentration where isolated cracks may nucleate favourably^2^ and their growth dynamics under subcritical loading may be strongly affected^3^. Ultimately, sustained microcrack initiation, multiplication and coalescence often results in a critical density of fractures whereby macroscopic rupture ensues. In this manner fracturing in heterogeneous materials is pervasive prior to failure as cracks propagate small distances between flaws and strain energy can be readily dissipated elastically^4^. In non-porous glasses, such elements of heterogeneity are lacking and the few crack nucleation sites available are typically nanoscopic in scale^5^. Therefore the crack propagation distance is relatively large and the strain energy stored must exceed the activation energy required for nucleation and propagation of fractures across the sample^2^. In such cases, little or no strain energy is released prior to rupture and fracturing is localised rather than pervasive. Thus more homogeneous materials possess a great propensity for highly catastrophic failure through rapid, unstable crack propagation associated with few precursory signals^1^,^6^.

In the Earth system, strain localisation and material failure control the timing of natural disasters. At volcanoes, the onset of an eruption is sometimes preceded by an acceleration in seismicity originating from the fracturing of rocks and formation of a conduit^7^,^8^; likewise eruptive transitions to explosions are also preceded by such characteristic seismic patterns^9^, that have been experimentally demonstrated to originate from magma failure^10^. In the case of landslides, a similar acceleration in seismicity may also be observed^11^,^12^. Thus empirical mechanistic models have been developed to describe the stress and strain rate extant upon failure of both porous rocks^4^ and magmatic suspensions^13^. Material deformation and failure in amorphous natural and synthetic materials is generally accompanied by accelerating precursory signals^3^,^8^,^10^,^14^,^15^,^16^,^17^,^18^,^19^. This acceleration represents the practical basis for the application of time-to-failure forecasting models^11^,^20^,^21^,^22^,^23^. During rock deformation, microcracking releases acoustic emissions (AE)^17^ prior to macroscopic failure. Their temporal, spatial and size distribution follow power laws^18^,^19^,^24^, similar to those observed in tectonic earthquake aftershock activity^25^ as well as in seismic precursors to volcanic eruptions^20^,^26^. Nevertheless, the wide range of materials in nature and especially the degree of material heterogeneity (at all scales) challenges our understanding of precursory signals leading to natural disasters^23^.

A great number of catastrophic events share similar characteristic accelerating trends in warning signals^23^ and are potentially describable via similar scaling laws^19^: rupture of engineering structures, natural catastrophes (such as great earthquakes, volcanic eruptions, landslides and avalanches), abrupt weather changes, some stock market crashes and even human parturition, amongst others. In many current models for precursory acceleration, the rate of seismic events 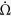 can be described by the Time-Reversed Omori Law (TROL)^25^.


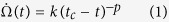


for which *k* is a scaling parameter, *p* parameterises the rate of acceleration, in turn dependent on the dominant crack mechanism^7^ and *t*_*c*_ is the critical time (corresponding to the time of system-sized catastrophic failure). This critical point is defined by a mathematical singularity as the quantity 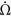 evolves toward infinity, providing a well-defined failure time. Equation 1 is directly analogous to the approach to a critical point in a second-order phase transition for the correlation length (size of the largest cluster or in our case the largest growing crack) as a function of temperature rather than time (also with a critical exponent analogous to *p*, which depends on the microscopic physics)^1^. It also corresponds to a general solution of Voight’s original empirical relation^21^ in the case that *α* ≠ 1 for which *p* = 1/(*α* − 1). The TROL is of widespread interest as a forecasting tool and has been extensively applied to material failure phenomena^7^,^8^,^9^,^10^,^11^,^12^,^21^,^22^,^26^,^27^. The TROL can be applied as an empirical relationship relating the acceleration of a physical observable to its rate under steady state conditions (stress or strain rate, temperature). In this context Equation 1 can be applied to any accelerating signal. In most applications to date, the equation is re-parameterised into a linear form


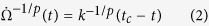


Equation 2 is commonly solved by linear regression, assuming a Gaussian error structure in inverse rate, and this is known in the literature as the Failure Forecasting Method (FFM). In a volcanic context, the most-commonly used parameter is seismic event rate, mainly because physical variables such as energy rate are subject to much more severe fluctuation. In application of the FFM *p* has been shown to decrease toward 1 as cracks grow^7^, so retrospective analyses of pre-eruptive seismic activity commonly assume *p* = 1. In some cases the Gaussian error assumption has been validated^28^, but this approach may yield a biased and imprecise solution if the assumption is not true^29^. In general it is more common in statistical seismology to assume a Poisson error structure, consistent with earthquake occurrence as a point process based on counting whole numbers of events^30^. In this case one can fit the rates to Equation 1 directly, using a Maximum Likelihood (ML) technique. Even so, it can be difficult to obtain representative uncertainties from single sequences or realisations, for example due to covariance between the error parameters^27^. In several realisations the ML method applied to the “full point” process has been demonstrated to provide (a) a more reliable estimate of the precision (random error) and (b) a more accurate solution, which reduces the potential for residual bias (systematic error) in forecasting the failure time^27^. The logarithm of the likelihood function *L* for the TROL is, in an interval (*t*_0_, *t*_1_), given by^27^


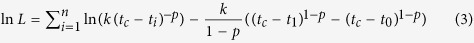


for *p* ≠ 1, and


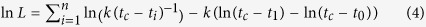


for *p* = 1.

The TROL is most commonly employed to describe the rate of pre-failure seismic events because it has a well-defined failure time. Other models have been proposed on theoretical or empirical grounds, including the exponential model 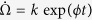 with *k* the pre-exponential scaling parameter and *ϕ* the rate constant; however, the failure time is not defined by the dynamics underlying the exponential model and failure forecasts using this model must be based on other metrics.

Here we experimentally test the hypothesis that the accuracy of failure forecasting improves as a function of the material heterogeneity using samples of variable quenched disorder, generated by the gas volume fraction (0–0.45) available during the synthesis (Fig. 1). This style of heterogeneity also provides a direct analogue for porous magma fragmentation^13^. Specifically we investigate the failure of variably porous silicate liquids undergoing the glass transition. Uniaxial compression of these porous materials was carried out at ~550 °C in the elastic, brittle regime by imposing a strain rate of 10^−3^ s^−1^ while monitoring AEs during deformation up to bulk failure (Fig. 2a). We find that highly porous suspensions are mechanically softer, and require less stress and strain to undergo failure than low-porosity ones. Their mechanical behaviour is well-described by the pore-emanating crack model^31^, demonstrating that their strength increases non-linearly with the heterogeneity index *H* (defined as the degree of disorder; see Methods Summary) and pore radius (Fig. 2b; the data appear to cut across the model contours as denser samples tend to have smaller pores). Deformation and failure is accompanied by AE energy release (Fig. 2c and Supplementary Figure 1) that accelerates more rapidly than an exponential function. The AE record reveals that the failure of low-porosity suspensions takes place via large (seismically energetic) fracture increments. Drastic fracture propagation upon failure releases the highest rate of AE energy, and this rate decreases systematically with increasing heterogeneity (Fig. 2d).

We applied the TROL using the ML method to the AE dataset in order to evaluate the forecasting performance quantitatively as a function of the heterogeneity index *H* (Fig. 3). The cumulative number of AEs released by fracturing is well-modelled by the TROL at all values of *H* (Fig. 3a). At low *H* actual failure occurs systematically before the predicted TROL singularity is reached, with this discrepancy increasing systematically as porosity decreases. We have also tested the alternate hypothesis that the acceleration may be exponential using a Bayesian Criterion Information (BIC; see Supplementary Information). The results indicate that the AEs released during the first stages of deformation generally follow an exponential trend. The TROL is strongly, non-linearly preferred over the exponential model when the entire dataset is used and importantly, as heterogeneity increases (Fig. 3b). On the other hand, as heterogeneity decreases we observe (1) fewer AEs (providing less advance warning), (2) a preference for the exponential acceleration model (making failure time harder to define) and (3) a sudden-onset singularity at the time of catastrophic failure. All of these elements combine to degrade the forecasting power significantly.

The associated forecasting error (defined as the absolute difference between the predicted failure time and the experimental failure time normalised by the deformation time) improves systematically with an increase in the degree of heterogeneity (Fig. 3c). This is most likely due to the fact that more heterogeneous materials act to inhibit dynamic fracture by crack arrest^3^ and/or by introducing a more heterogeneous stress field^6^ (consistent with the quasi-static theories used to derive Equation 1). In the more homogeneous materials failure results in an abrupt run-away instability that occurs before the forecast singularity is reached. As a consequence our systematic forecast error is smaller (the predicted failure time is more accurate) when applied to more heterogeneous materials containing gas volume fractions >0.2, whereas at gas volume fractions <0.2, the error in the predicted failure time can be >100% of the deformation time. In operational terms this would present a serious challenge, for example in forecasting the probability of an eruption during a period of unrest.

Complementary statistical analysis of the AE signals following the seismic Gutenberg-Richter (G-R) *b*-value (*i.e.*, the slope of the log-linear frequency-magnitude relationship) indicates that cracking occurs on a broad range of scales as deformation proceeds. The AE *b-*value is strongly controlled by the degree of heterogeneity, confirming early observation^15^ (Supplementary Figure 3). The temporal evolution of the *b*-value with stress is harder to examine, due to the small number of events. In Supplementary Figure 3 this is examined in a coarse way by splitting the data set into two halves, one early and one later. In general the *b*-value for materials with large heterogeneity tends to decrease dramatically from >2 to ~1, well above the level expected from the estimated random error (plotted as error bars). This is interpreted as initially pervasive microscopic fractures coalescing into macroscopic ones^32^ and the deformation localising on the eventual fracture plane. In contrast, the *b-*value of less porous material remains around low values of 0.5–1 throughout, suggesting a high degree of localisation throughout^32^. This is consistent with there being fewer nucleation sites for the low-porosity material. The data presented here is not sufficient to distinguish between models with (a) simple G-R behaviour with variable *b*-value and (b) an exponentially-truncated G-R model with constant *b*-value and variable correlation length (*i.e.*, the size of the largest fracture). The latter model and a smooth acceleration in event rate for the heterogeneous samples are however both consistent with the behaviour expected of a second-order phase transition at the critical point^1^. On the other hand, the sudden-onset instability for the more homogeneous samples is more reminiscent of a first-order phase transition. Numerical simulations should be employed in future to explore this transition from first- to second-order more formally.

Understanding the potential drawbacks and limitations of the FFM is an essential aspect of their responsible application to hazard assessment and risk mitigation. Previous studies have evaluated its statistical performance applied to natural, experimental and synthetic datasets^27^,^29^ but to date no study (to the best of our knowledge) has assessed its efficacy as a function of material properties and the trade-off between quasi-static and dynamic effects at the system size. At volcanoes, successful forecasting is as yet sporadic and requires the sometimes laborious classification of volcano-seismic signals. While the onset of magma extrusion due to continued fracturing towards the Earth surface has been retrospectively successfully forecast or ‘hind-casted’^7^,^26^, this is a necessary but not sufficient criterion for operational or real-time forecasting. In the case of fracturing during magma ascent, seismicity is most likely triggered by fracture propagation in the weakest, most porous parts of the magmatic column. In cases where low-porosity, fine-grained rock or glassy obsidian undergoes fracturing initiated from fewer flaws, we expect to encounter a poor resolution of failure. Such a variable failure forecasting power should equally well apply to the prediction of explosive eruptions for magmas erupting with different porosities.

These results shed new light onto the basic physical mechanisms responsible for inaccuracy of time-to-failure forecasting laws, especially in the context of volcanic eruptions. In scenarios where magma ascent timescale is very brief and shorter than that of the seismic unrest, strong deviations from the ideal preparatory fracturing behaviour must be expected. Nevertheless, our results suggest that adaptation of material failure forecasting methods with heterogeneity-based mechanistic constraints, in particular accounting for the bias revealed in Fig. 3c, could in principle improve the predictability of volcanic events even in cases when little warning is available.

## Methods Summary

### Sample preparation

The suite of samples was fabricated by viscous sintering under no external applied stress^13^. We used industrial soda-lime silica beads (Spheriglass^®^ A-glass microspheres 1922, 2024 and 2530, Potters Industries Inc.) with well-constrained chemical and physical properties such as the calorimetric glass transition interval and the viscosity-temperature dependence. This material is also chemically stable and does not crystallise or degas at the experimental conditions. We systematically packed glass beads in alumina ceramic crucibles (44 mm diameter and 75 mm height) and heated them at 10 °C min^−1^ to an isotherm above the glass transition at which the melt viscosity is 10^8.32^ Pa.s. Viscous sintering took place during dwells of 1 to 32 hours and the samples were cooled down at a slower rate of ~5 °C min^−1^ to avoid induced thermal cracks. The densified products were finally drilled out from the crucibles to sample cores of 25 mm diameter by 50 mm height. The gas volume fraction in the suite of cores was calculated from the density of the bulk sample and the powdered glass density as measured after sintering.

### Heterogeneity quantification

Structural heterogeneity or disorder is evaluated from the normalised difference between the volumes of both phases (*i.e.*, glass *V*_*glass*_ and gas *V*_*pores*_). These volumes can be calculated from the measured total porosities and, hence, a heterogeneity index *H* being directly linearly proportional to gas volume fraction *ϕ*_*g*_. We define the order parameter *Q* as


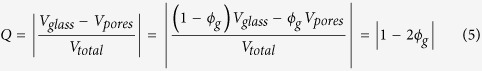


where *V*_*total*_ is the bulk volume of the sample. It follows that at *ϕ*_*g*_ = 0 (pore-free) or *ϕ*_*g*_ = 1 (no solid phase), *Q* = 1 (*i.e.*, perfect order), and that at *ϕ*_*g*_ = 0.5, *Q* = 0 (*i.e.*, maximum disorder). The heterogeneity index (or disorder index) is thus calculated following *H* = 1 − *Q.*

### Mechanical testing

A series of uniaxial compression tests was performed using a <300 kN press, which is equipped with a split furnace (≤1100 °C) surrounding the pistons (in order to simulate magma deformation in the upper volcanic conduit). The porous glasses were loaded to failure at a constant strain rate of 10^−3^ s^−1^ and ~550 °C in order to recover purely elastic behaviour. The cooler ends of the pistons were equipped with AE broadband transducers (of 125 kHz central frequency), thus used as waveguides for AEs released during fracturing events and catastrophic sample failure. The AE signal was transferred using buffered 40 dB preamplifier to a data acquisition system (Richter system, Applied Seismology Consultants), which recorded AE voltage data continuously at a sampling rate of 20 MHz.

### Microseismic processing

AE event onsets were triggered and automatically picked from continuous acoustic streams using an adaptation of the standard autoregressive-Akaike-Information-Criterion (AR-AIC) picker. The multi-step algorithm consists of (1) detection of the P-phase using a standard STA/LTA detector, (2) de-noising of the acoustic signal and (3) AIC computation (the minimum indicates the first arrival time). The STA and LTA windows were set to 1 and 20 ms respectively and the STA/LTA threshold to 2. The amplitude in dB and energy in nJ of each single event was also computed (based on a resistance reference standard value of 10 kΩ). These pre-failure catalogues of acoustic events were used as the basis for failure forecasting.

### Failure forecasting

Following the procedure described in detail in reference 27, we applied the TROL to catalogues of acoustic events in order to retrospectively forecast failure. This law has three free parameters (*k*, *p* and *t*_*c*_) to adjust since they are not known *a priori*. The ML method has been shown to provide statistically stable and repeatable estimates of these parameters^27^. Additionally this method uses the timings of individual AE events rather than event rates determined in equally spaced bins (as is commonly the case when applying the standard FFM). The ML solution is found by minimizing the negative log-likelihood function using a downhill simplex algorithm. The forecasting window was restricted to 90% of the known failure time. Uncertainties on the fitted parameters require prior constraint to be reliably computed such that this precludes the estimation of meaningful error bars on the forecasted failure times.

## Additional Information

**How to cite this article**: Vasseur, J. *et al.* Heterogeneity: The key to failure forecasting. *Sci. Rep.* **5**, 13259; doi: 10.1038/srep13259 (2015).

## Supplementary Material

Supplementary Information

## Figures and Tables

**Figure 1:**
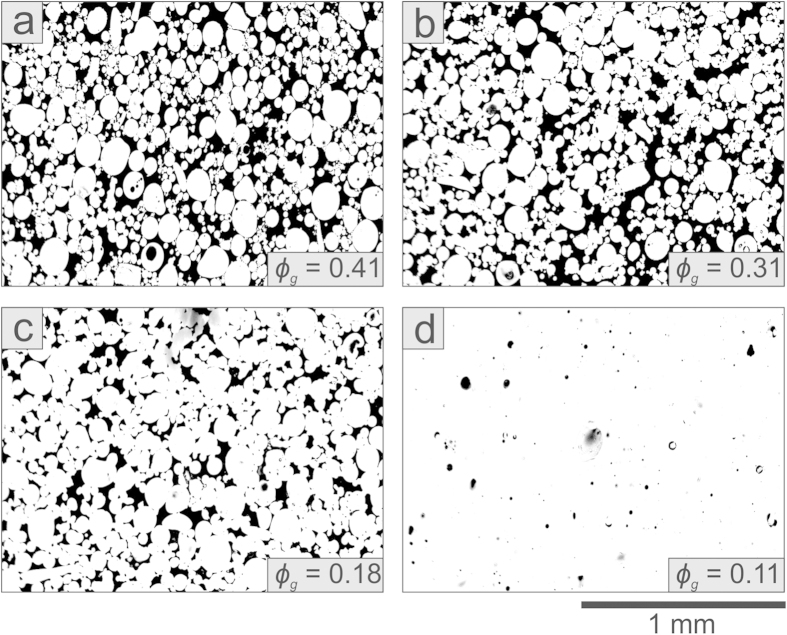
Structural heterogeneity in the sintered glass samples. Scanning Electron Microscopy (SEM) images in binary false-colour of thick sections of synthetic samples sintered at 650 °C for incremental times. These porous glasses feature a wide range of total porosity – from high (**a**) to low (**d**) – estimated by the gas volume fraction *ϕ*_*g*_. Black represents the pores and white the glass matrix.

**Figure 2:**
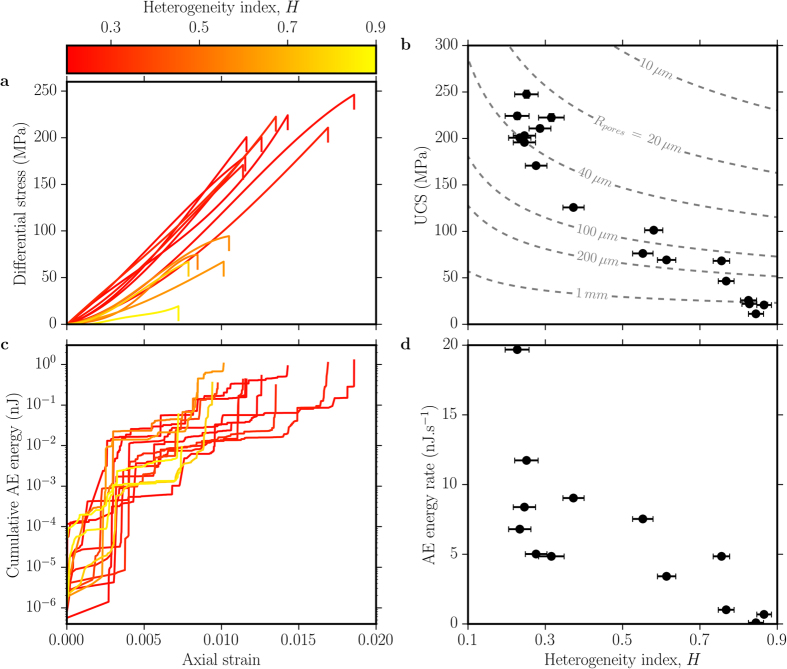
Acoustic-mechanic response of porous glasses deformation. (**a**) Uniaxial elastic loading and the resultant stress-strain build-up leading to bulk failure (stress drop) at ~550 °C and 10^−3^ s^−1^. (**b**) Uniaxial Compressive Strength (UCS), as measured by the peak stress at failure, against the heterogeneity index *H.* Displayed are the predicted isopore lines for different radii (dashed grey lines) from which crack initiate in the pore-emanating crack model^31^ (see text). (**c**) Cumulative AE energy released during deformation until sample failure (**d**) Heterogeneity-dependence of the AE energy rate upon failure of the specimens. (**a**,**c**) are colour-coded from low to high heterogeneity samples.

**Figure 3:**
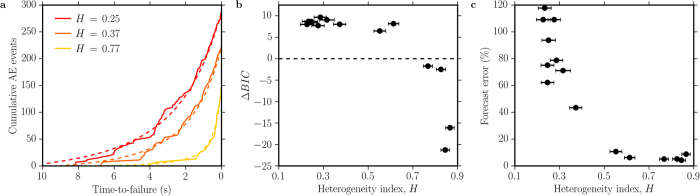
Heterogeneity influences on material failure forecast. (**a**) Cumulative AE events (solid lines) and their ML best-fit curves (dashed lines). (**b**) Δ*BIC* (*BIC*_*TROL*_ − *BIC*_*Exp*_) displays a marked preference of the TROL over the exponential model as heterogeneity increases. (**c**) Heterogeneity-dependence of the forecast error, defined as the absolute difference between the predicted failure time and the experimental failure time normalised by the deformation time.
