# Supplementary material for: Heterogeneity: The key to failure forecasting
Source: Sci Rep. 2015 Aug 26;5:13259. doi: 10.1038/srep13259 (PMC4549791; doi:10.1038/srep13259)
Supplement: Supplementary Information [file srep13259-s1.pdf]

Supplementary Information of *Heterogeneity: The key to failure forecasting*

Jérémie Vasseur[1], Fabian B. Wadsworth[1], Yan Lavallée[2],

Andrew F. Bell[3], Ian G. Main[3], Donald B. Dingwell[1]

[1] Earth and Environmental Sciences, Ludwig Maximilian University, Munich, Germany

[2] Earth, Ocean and Ecological Sciences, University of Liverpool, Liverpool, United Kingdom

[3] School of Geosciences, University of Edinburgh, Edinburgh, United Kingdom

1. AE energy released

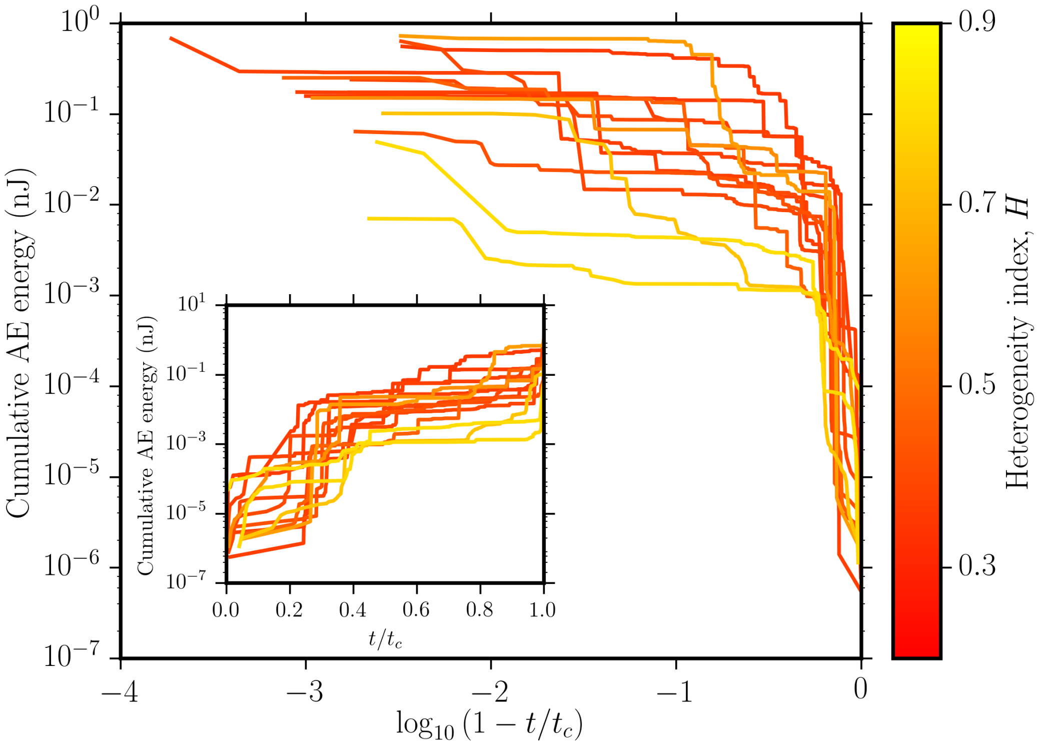

Supplementary Figure 1 – **Acoustic response of porous glasses deformation up to failure.** Cumulative AE energy released during deformation plotted in a log-log and a semi-log (inset) space and colour-coded from low to high heterogeneity samples. One can observe the overall tendency to behave according to a power law towards failure.

2. Model comparison

We undertook a comparative analysis, testing how well different models, namely a power law (TROL), an exponential (Exp) and a constant rate (CR; the event rate $\dot{\Omega}$ is set equal to a constant c) model, explain the observed evolution of AE rate with time. We used the Bayesian Information Criterion (BIC) to quantify the relative performance of different models. This statistical tool is based on the likelihood of the observation given the model, with a weighting favouring the model with fewer parameters. The BIC[1] is given by

$$BIC = -2\ln(L) + N_p \ln(N_o) \quad (1)$$

where L is the likelihood of the observations given the model, N_p is the number of free parameters and N_o is the number of observations. When making an inference, the preferred model is more likely to have the lower BIC. Therefore, calculating the positive difference ΔBIC between two models helps discriminate the preferred model. Here we computed these differences (*i.e.*, $\Delta BIC = BIC_{TROL} - BIC_{Exp}$ and $\Delta BIC = BIC_{TROL} - BIC_{CR}$ for the AE dataset continuously from 50 to 100% of the sequences, such that when ΔBIC becomes negative it indicates a strong statistical preference for the power over the other models (Supplementary Figure 2).

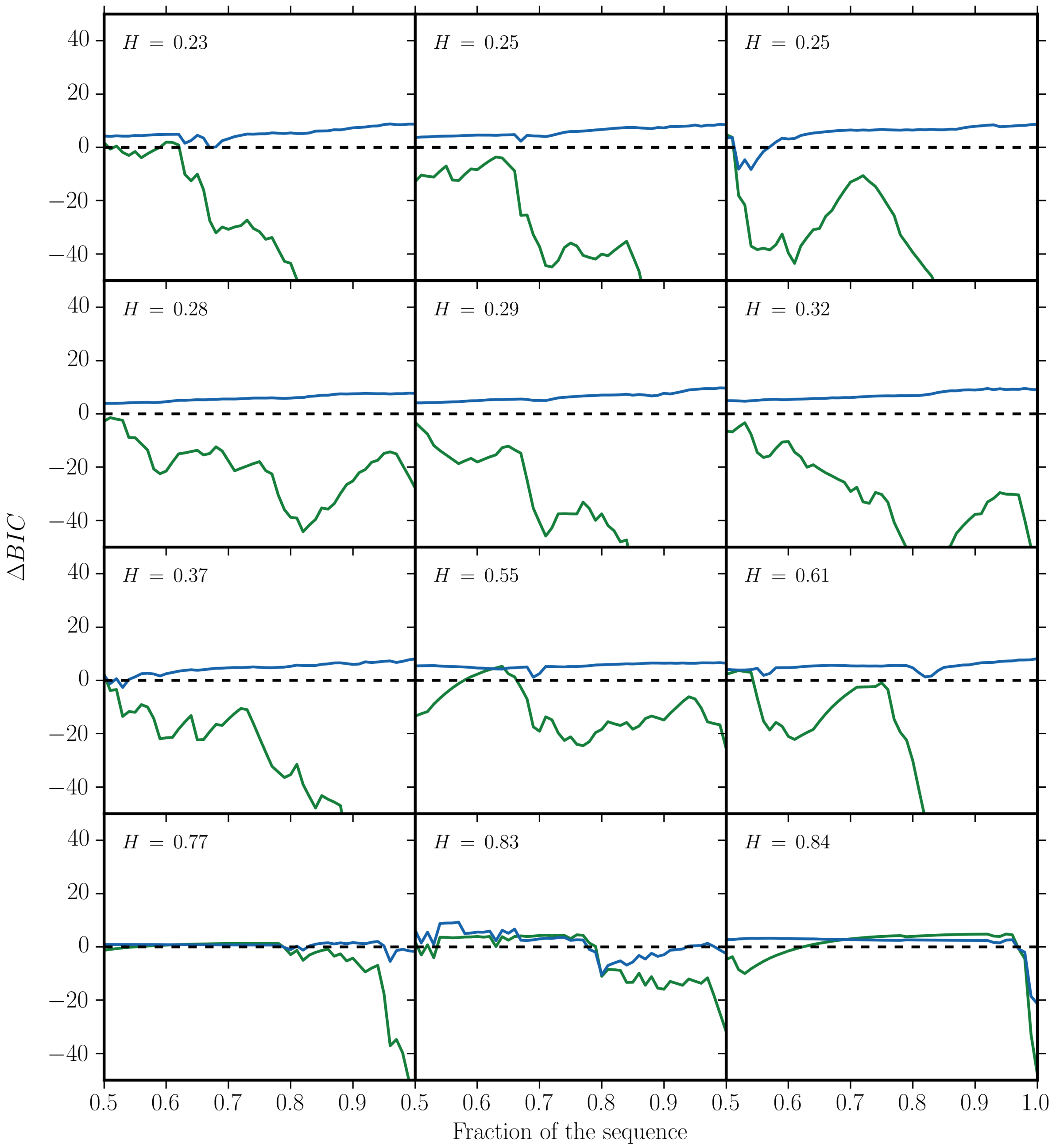

Supplementary Figure 2 – **Statistical comparative analysis of model performance with material heterogeneity.** Comparison of the ΔBIC between the TROL and exponential model (solid blue line) and between the TROL and constant rate model (solid green line) as a function of the time fraction to achieve failure, which corresponds to 1. Negative values of ΔBIC suggests that the TROL is preferred over the other models. A marked preference for the TROL over the exponential model as heterogeneity is increased. $\Delta BIC = 0$ is indicated as a horizontal dashed black line.

3. AE *b*-value

The frequency-magnitude data for the AEs is consistent with a Gutenberg-Richter distribution (Supplementary Figure 3). The Gutenberg-Richter *b*-value was determined for AE events above the completeness magnitude using the maximum-likelihood estimate[2]. Completeness magnitude is taken as the higher of the two values determined by the maximum curvature and *b*-value stability methods[3]. Sensitivity analysis showed that the key *b*-value results were robust to different completeness magnitude estimation methods, and to small uncertainties in the completeness magnitude.

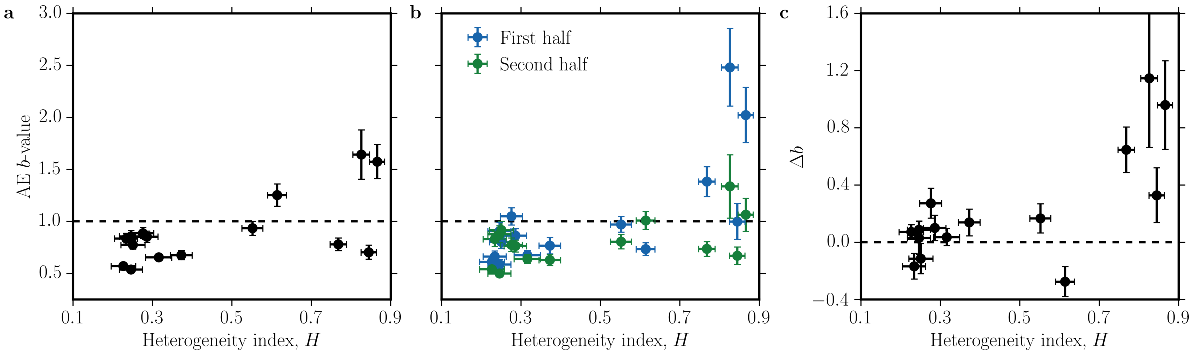

Supplementary Figure 3 – **AE *b*-value analysis and fracture mechanisms in porous glasses.** The *b*-values were determined **a**, for the complete experimental AE record, and **b**, for the first and last halves of the AE events acquired in each experiment (with completeness magnitudes being calculated separately for each half of the data). The AE *b*-values lie in the range 0.5-1.0, except for samples with higher degrees of heterogeneity, where two experiments show *b*-values of ~1.6. **c**, Difference in *b*-values $\left(\Delta b = b_{initial} - b_{final}\right)$ shows that the *b*-value generally decreases as failure approaches, indicating that there is an increase in the relative proportions of large to small AE events that accompany increasingly macroscopic fracturing events. A *b*-value of 1 is indicated as a horizontal dashed black line.

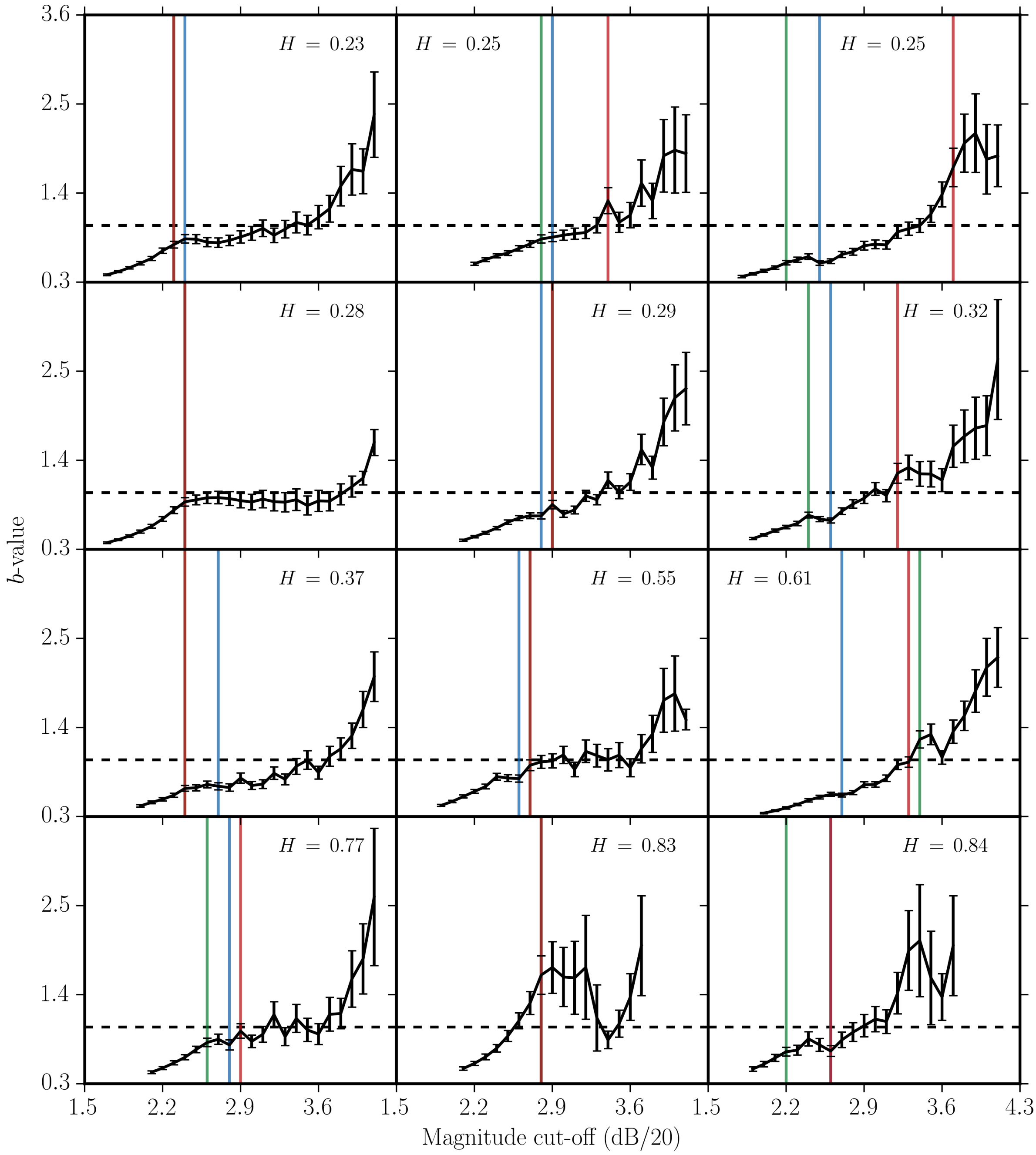

Supplementary Figure 4 – **AE *b*-value and completeness magnitude analysis with material heterogeneity.** AE *b*-value determined using the maximum-likelihood estimate (solid black line) as a function of magnitude cut-off. Completeness magnitude computed using the maximum curvature, the *b*-value stability and the goodness-of-fit test methods are represented by the vertical blue, green and red lines respectively. A *b*-value of 1 is indicated as a horizontal dashed black line.

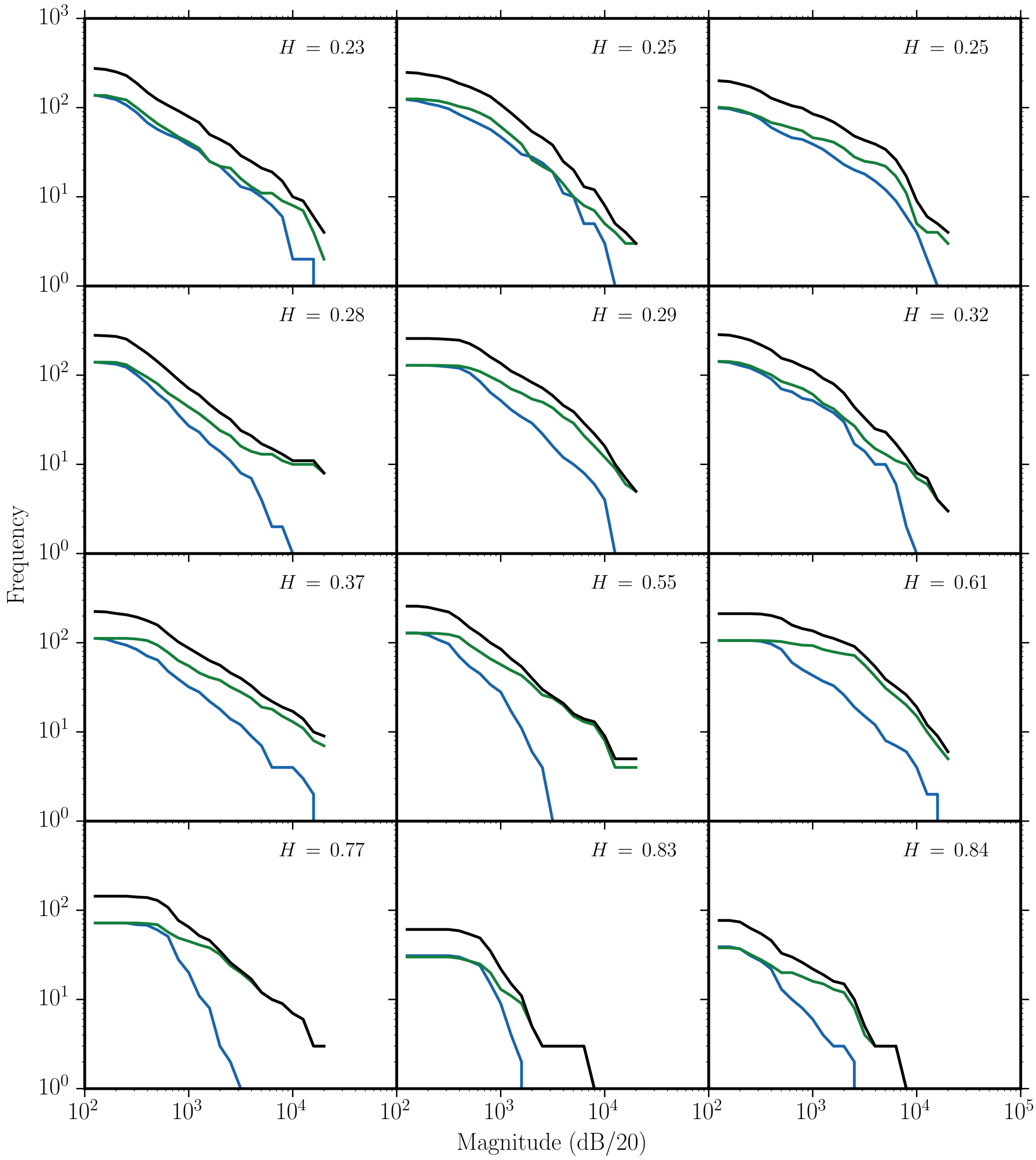

Supplementary Figure 5 – **Frequency-magnitude analysis with material heterogeneity.** Comparison of frequency-magnitude data obtained for the first half of the sequence (solid blue line), the second half of the sequence (solid green line) and the full sequence (solid black line). One can observe the overall tendency to follow a Gutenberg-Richter distribution.

4. Failure forecast

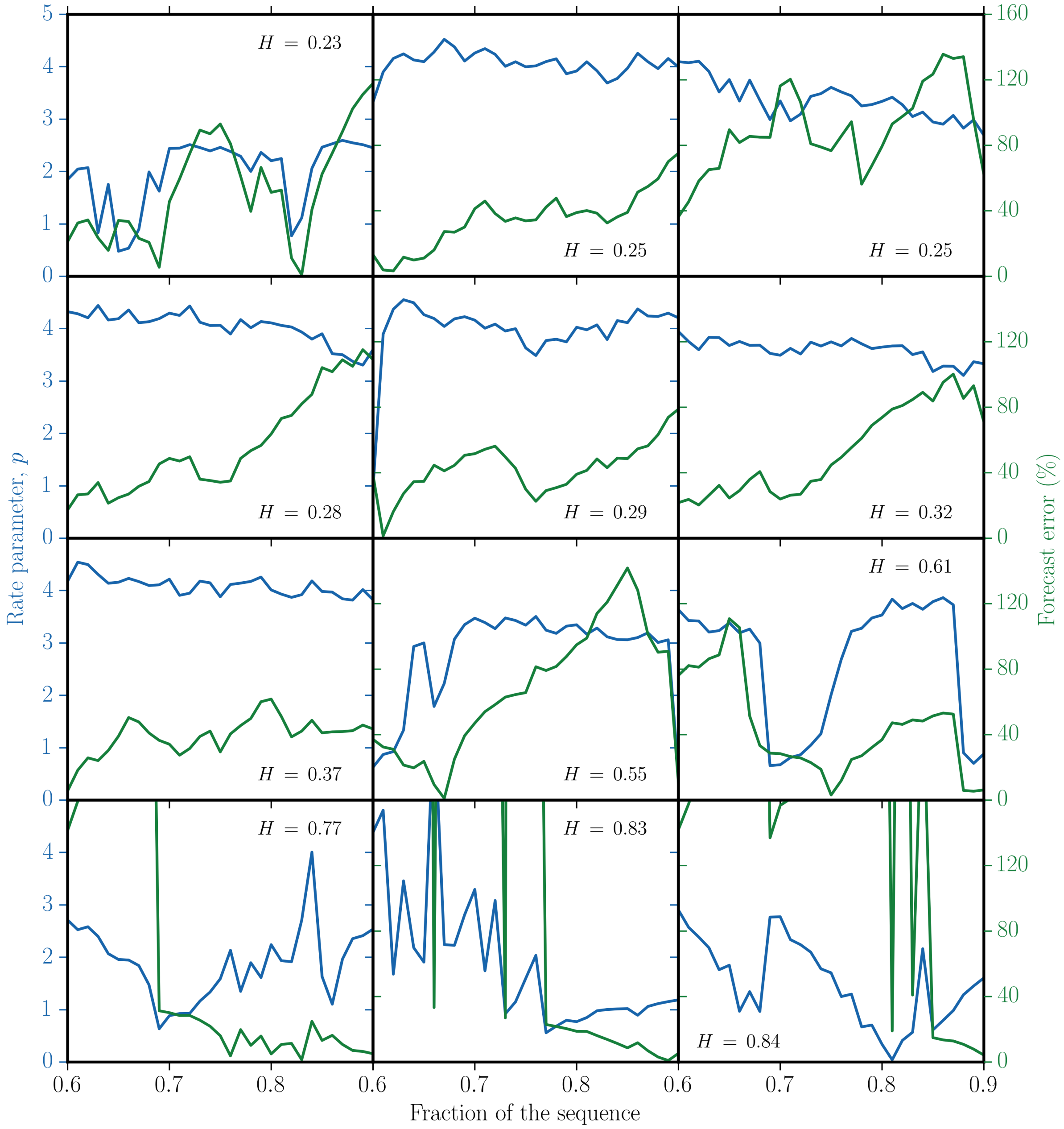

Supplementary Figure 6 – **Failure forecast analysis with material heterogeneity.** Comparison of the TROL rate parameter (solid blue line) and the forecast error (solid green line) as a function of the time fraction to achieve failure, which corresponds to 1. A marked decrease of the rate parameter towards lower values and an improvement of the forecast error as heterogeneity is increased.

5. References cited

1. Kass, R. E. & Raftery, A. E. Bayes factors. *J. Am. Stat. Assoc.* **90**, 773–795 (1995).
2. Aki, K. Maximum Likelihood Estimate of b in the Formula logN=a-bM and its Confidence Limits. *Bull. Earthq. Res. Inst.* **43**, 237–239 (1965).
3. Mignan, A. & Woessner, J. Estimating the magnitude of completeness for earthquake catalogs. *Community Online Resource for Statistical Seismicity Analysis*, 1–45 (2012).
